# A Safe-by-Design Approach to “Reef Safe” Sunscreens Based on ZnO and Organic UV Filters

**DOI:** 10.3390/antiox11112209

**Published:** 2022-11-09

**Authors:** Mattia Battistin, Paolina Pascalicchio, Beatrice Tabaro, Dritan Hasa, Alessandro Bonetto, Stefano Manfredini, Anna Baldisserotto, Alessandro Scarso, Paola Ziosi, Andrea Brunetta, Fabio Brunetta, Silvia Vertuani

**Affiliations:** 1 Kalis Srl, Via Caodevilla 38, 31040 Pederobba, Italy; 2 Faculty of Medicine, Pharmacy and Prevention, Department of Life Sciences and Biotechnology, Univesity of Ferrara, Via L. Borsari 46, 44121 Ferrara, Italy; 3 Department of Chemical and Pharmaceutical Sciences, University of Trieste, Piazzale Europa 1, 34127 Trieste, Italy; 4 Department of Environmental Sciences, Informatics and Statistics, University Ca’ Foscari Venice, 30123 Venezia, Italy; 5 Department of Molecular Sciences and Nanosystems, University Ca’ Foscari Venice, Via Torino 155, 30172 Venezia, Italy; 6 Ambrosialab Srl, Via Mortara 171, 44121 Ferrara, Italy

**Keywords:** cosmetic, ZnO, sunscreen, photocatalysis, superoxide anion, hydroxy radical, Diethylamino Hydroxybenzoyl Hexyl Benzoate, Ethylhexyl Triazone

## Abstract

In recent years, the issue of coral bleaching has led to restrictions in some tropical locations (i.e., Palau, Hawaii, etc.) on the use of some organic UV sunscreen filters, such as oxybenzone and ethyl hexyl methoxycinnamate. In contrast, ZnO is considered safe for marine environments and thus is often used without considering its photocatalytic and oxidative activities related to the generation of O_2_^•−^ and HO^•^. Moreover, ZnO needs to be used in combination with other filters to reach higher protection factors. Thus, the study of its interaction with formulations and with organic filters is important in sunscreen technology for the development of safer by-design products. In this work, the photocatalytic activity of zinc oxides with different surface areas (30, 25 and 9 m^2^/g) and their interaction with selected organic sunscreen filters were investigated. In particular, the ZnO photocatalytic kinetics were studied following the photodegradation of Acid Blue 9 (AB9) observing a first-order reaction with a chemical regime. Our evaluations of the selective inhibitions by h_vb_^+^ and HO^•^ demonstrated a substantial predominance of the hydroxide radicals in the expression of the photocatalysis, a trend that was also confirmed by the irradiation of ZnO in an ethanolic solution. Indeed, the formulations containing both ZnO and organic filters defined as “safe” for coral reefs (i.e., Diethylamino Hydroxybenzoyl Hexyl Benzoate, DHHB, and Ethylhexyl Triazone, EHT) showed a non-negligible photocatalytic oxidation and thus the combination was underlined as safe to use.

## 1. Introduction

From an industrial point of view, ZnO has definite advantages as a UV sunscreen filter when compared to the better known TiO_2_, showing a wider absorption spectrum with a threshold wavelength of 425 nm. The study of ZnO’s interaction with light radiation has attracted great interest especially in the recent years due to the exponential growth of cosmetic products that have a natural and sustainable connotation (CPNSC). The photocatalytic activity of ZnO has been well known and well documented for a long time [1]. However, its use as a UV filter ingredient is now recognized and allowed even in nations that strongly regulate the use of sunscreens in order to preserve the integrity of coral reefs [2,3]. Ultraviolet (UV) organic filters found in personal care products are under a magnifying glass because of concerns that they are an emerging toxic threat to reef organisms [4]. Furthermore, the use of inorganic filters is gaining increasing attention due to their photocatalytic activity [5].

**Figure 1:**
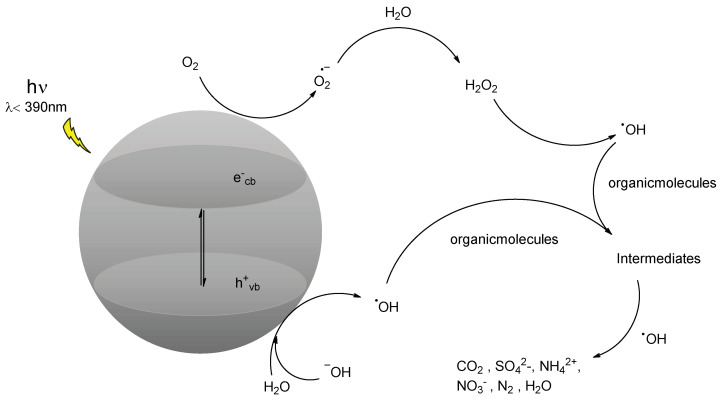
Diagram of reactions involving ZnO photocatalysis. Adapted from [6].

Upon irradiation, electrons in the valence band (VB) are promoted to the conduction band (CB), (Figure 1). The hole–electron pairs can then recombine with a release in heat, but they can also interact with other molecules, such as those of adsorbed water, to provide hydroxylic radicals (hole, VB) or superoxide ions by reduction (electrons, CB). The reactivity of hydroxyl radicals leads to the formation of intermediates following the interaction with organic species dissolved in water [6].

(1) $$ZnO+h\nu \to e^{-}+h^{+}$$

(2) $$e^{-}+h^{+}\to heat$$

(3) $$h^{+}+H_{2}O_{ads}\to \;OH_{ads}+H^{+}$$

(4) $$h^{+}+OH_{ads}{}^{-}\to {}^{•}OH_{ads}$$

(5) $$e^{+}+O_{2}\to O_{2}^{•-}$$

(6) $$O_{2}^{•-}+HO_{2}^{•}+H^{+}\to H_{2}O_{2}+O_{2}$$

(7) $$O_{2}^{•-}+organicmolecules\to organicmolecules-OO^{•}$$

(8) $$OH_{ads}+OM\to Intermediates\to P$$

Given the current health and state of coral reef ecosystems caused by present climate trends, it is important to consider the additional risks posed by exposure to these chemicals in order to mitigate further degradation. Thus, continuing our efforts toward the development of safer and sustainable sunscreen formulations within the frame of the European project SBD4Nano (https://www.sbd4nano.eu, accessed on 30 October 2022), we started the present study using a safe-by-design approach. As clearly defined by van de Poel [7], “Safe-by-design (SbD) aims at addressing safety issues already during the R&D and design phases of new technologies. SbD has increasingly become popular in the last few years for addressing the risks of emerging technologies such as nanotechnology and synthetic biology”. SbD can be envisaged as a risk management strategy that addresses safety by designing measures using a self-contained safety or a risk assessment approach, taking into account risks in the early design phase. The remediation of damages caused by a hastily developed product involves much greater costs and more time than product design strategies that predict possible adverse behavior due to the interaction between components of the formula or between components, humans, and/or the environment. Indeed, sunscreens are not just “normal” skin products, because UV filters actively interact with UV radiation (such as in a reactor) and become able to interact with other sunscreen UV filters present in formulations.

## 2. Materials and Methods

### 2.1. Chemicals

Zinc oxide samples were obtained from EverZinc (Loncin, Belgium). The Acid Blue 9 molecule “AB9” (≥99%, CAS number 3844-45-9) used for photocatalitic activity detection was obtained from Farmalabor (Milan, Italy). The analysis of sunscreen degradation by ZnO activity was carried out by solubilizing the organic sunscreen molecules in water. Both surfactant mixture (Eumulgin^®^ HPS, formed by a mixture of Coceth-7–CAS number 61791-13-7-, PPG-1-PEG-9 Lauryl Glycol Ether–CAS number 154248-98-3-, and PEG-40 Hydrogenated Castor Oil–CAS number 61788-85-0-) and the sunscreen molecules Diethylamino Hydroxybenzoyl Hexyl Benzoate (DHHB, ≥99%, CAS number 302776-68-7) and Ethylhexyl Triazone (EHT, ≥99%, CAS number 88122-99-0) were provided by BASF (Ludwigshafen am Rhein, Germany). The solvents for HPLC analysis, ethanol (EtOH, absolute CAS number 64-17-5), acetonitrile (ACN, ≥99.9%, CAS number 75-05-8) and water (≥99.9%, CAS number 7732-18-5) were obtained from Merck KGaA (Darmstadt, Germany).

### 2.2. Determination of Photocatalytic Activity and Kinetic Studies

#### 2.2.1. Evaluation of Photocatalytic Activity of Different Zinc Oxides

**Figure 2:**
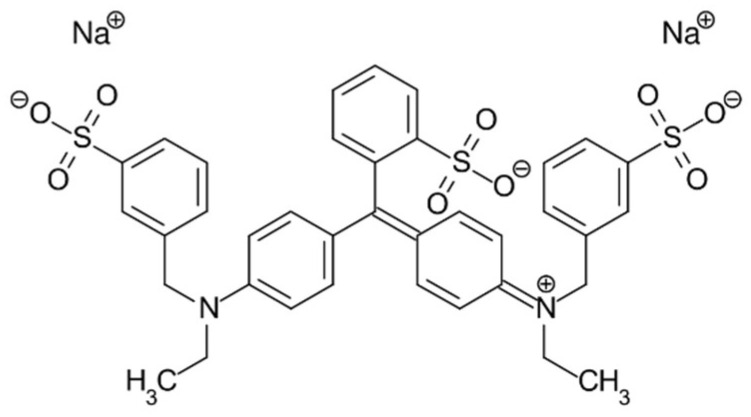
Structural formula of Acid Blue 9 (AB9).

To verify ZnO’s photocatalytic activity, a solution of Acid Blue 9 85% (10^−5^ M) was prepared in a 500 mL flask using water as solvent. Acid Blue 9 (Figure 2) is a water-soluble synthetic food dye used in analyses as an indicator and shows a maximum absorption between 629 and 638 nm (UV–Vis V-730 JASCO International Co. Ltd., Tokyo, Japan).

A calibration curve was then created with 0.8, 4.0, 8.0, 24, 40 and 80 ppm standard solutions. The tests were performed by adding 0.1 g of ZnO to 40 mL of 80 ppm AB9 solution. After 20 min, a homogeneous mixture was obtained and irradiation with UV lamp was performed at 354 nm (Thermo Fisher Scientific, Waltham, MA, USA). In order to exclude the contribution related to adsorption, two distinct solutions were prepared, one irradiated and the other stored in the dark. The analyses were carried out using a spectrophotometer after centrifugation at 5000 rpm for 10 min. Samples were taken at intervals of 5, 10, 20 and 30 min.

#### 2.2.2. Degradation of Organic Species with ZnO-Based Photocatalyst: Determination of Main Reactive Species

In order to verify the photocatalysis mechanism of ZnO and to distinguish the reactive species involved in the reactions, scavengers were alternately added to the solution to investigate the importance of radicals since these scavengers selectively react with them, forming stable or persistent intermediates.

For example, alcohols, such as isopropanol, methanol and tert-butanol, are commonly used to investigate the HO^•^-mediated mechanism. In addition, the direct oxidation of these alcohols by h_vb_^+^ may be negligible because of their very low affinity with the surface of ZnO.

The iodide ion is an excellent scavenger that reacts with the h_vb_^+^ and HO^•^ adsorbed, because h_vb_^+^ can be easily captured by I^−^ (being an electron donor). The oxidation of I^−^ in aqueous solutions by superficial HO^•^ can also occur [8].

In this work, tert-butanol and KI were used as scavengers. The following samples were prepared:

1. Solution without scavenger;
2. 10 mM tert-butanol solution;
3. 100 mM tert-butanol;
4. 0.4 mM KI solution;
5. 4 mM KI solution.

The analysis was carried out and we weighed 0.1 g of ZnO in 40 mL of AB9 solution. The samples were placed under UV irradiation and detections were performed at 5 min, 10 min, 20 min and 30 min.

#### 2.2.3. Determination of the Photocatalytic Activity of ZnO against DHHB and EHT

The photocatalytic activity of ZnO against DHHB and EHT organic filters was evaluated by HPLC (LC-4000 JASCO International Co. Ltd., Tokyo, Japan) titration of the earthenware filters following exposure to UV light radiation. We proceeded by working in two different environments, one with water solvent and the solubilizer mixture of Coceth-7, PPG-1-PEG-9 Lauryl Glycol Ether and PEG-40 Hydrogenated Castor Oil (HPS), and the other with ethanol as solvent. In the first case, 0.01 g of DHHB and ETH was carefully weighed, then 1.0 g of HPS was added and the solution was heated to 50 °C under continuous mixing. Once solubilization was reached, 50 mL of water was added, followed by 0.1 g of ZnO. The mixing proceeded for 10 min, without exposure, giving the system time to balance. Afterwards, the irradiation with UV lamp was started. Titration took place at intervals of 5, 10, 20, 30 and 60 min as well as 6 h and 24 h. At any time, 3 mL of the solution was taken, centrifuged for 10 min at 6000 rpm, then filtered by a 0.45 μm filter and finally diluted 10 times with HPLC-grade water before injection into the column. The same steps were followed in the case of ethanol as a solvent, except the solubilizer was not used. In this case, the pre-entry dilution in the column took place with HPLC-grade ethanol.

### 2.3. Particle Size Determination

About 10 mg of each zinc oxide’s dry powder was dispersed separately in 50 mL of MilliQ water filtered at 0.45 µm and sonicated with a probe in an ice bath at 200 W for 5 min (in 80% pulsed mode). After the dispersion, 1 mL of sample was transferred into a polystyrene cuvette for the particle sizing. The analysis was conducted by means of dynamic light scattering (DLS) (Ultra Dynamic Light Scattering, Zetasizer Ultra, by Malvern Panalytical Ltd., Malvern, United Kingdom), at 25 °C, acquiring the signal at 90 °C for 2 min.

### 2.4. Titration of Organic Filters

An HPLC method for UV organic filter determination was developed. The method was a gradient and used a standard C18 column (Mediterranea, C18, 4.6 mm × 150 mm, 5 μm). The solvents were CH_3_CN/H_2_O 90/10 (Solvent A) and EtOH 100 (Solvent B) with increasing concentration from 50/50 Solvent A/Solvent B to 100% Solvent B in 20 min. The concentration of Solvent B was kept at 100% for another 3 min, and then returned to the conditions of 50/50 Solvent A/Solvent B for the remaining 2 min. The flow was 1 mL/min. The chromatogram was acquired at 330 nm. The elution of DHHB took place at 10 min, while that of ETH was at 19 min. The standards were prepared from a 0.025 M DHHB and 0.012 M ETH solution to obtain the different concentrations of 0.5, 1, 5, 10, 30 and 50 ppm.

## 3. Results

### 3.1. Determination of Photocatalytic Activity and Kinetic Studies

#### 3.1.1. Photocatalytic Activity of Different Zinc Oxides

The photocatalytic activity of ZnO was determined by monitoring the degradation of the Acid Blue 9 molecule following irradiation at 365 nm, the wavelength relative to the maximum absorbance of ZnO. Degradation monitoring took place at time intervals of 5, 10, 20 and 30 min. For each analyte, two conditions were evaluated: one with irradiation and one with simple mixing in the dark to exclude the component related to the surface adsorption of the dye on the ZnO. Three types of ZnO with different particle sizes and surface areas (30, 25 and 9 m^2^/g) were evaluated and named Z30-Ps, Z25-Ps and Z9-Ps, respectively. All the reaction kinetics and the value of the kinetic constant (k_obs_) were established. Prior to irradiation, the adsorption of the dye was evaluated to avoid interference with the data analysis. The generic reaction was hypothesized as follows (Equation (9)):

(9) $$AB9\;\overset{ZnO,\;k_{obs}}{\to }\;P$$

**Figure 3:**
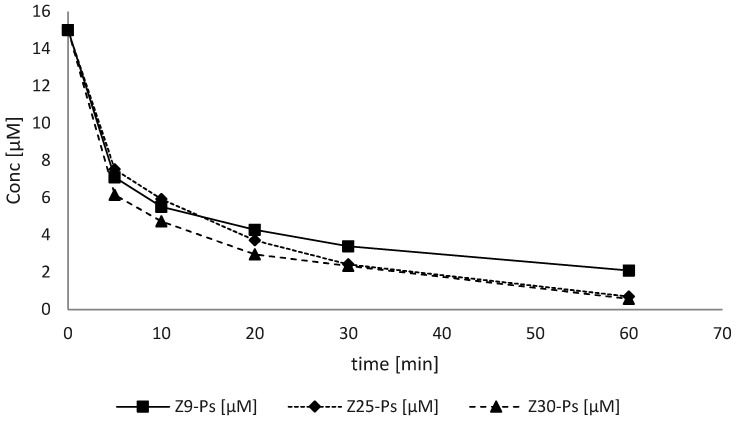
Trend of the concentration of dye over time following irradiation with UV radiation. ZnO-induced photocatalysis of different particle sizes.

The reaction order was graphically verified (Figure 3) by assuming a first- and second-order reaction:

For a first-order kinetic, the rate equation is described by Equation (10):

(10) $$r_{AB9}=-k_{obs}\left[AB9\right]$$

from which Equations (11) and (12) are derived:

(11) $${\left[AB9\right]}_{t}={\left[AB9\right]}_{0}e^{-k_{obs}t}$$

(12) $$\ln{\left[AB9\right]}_{t}=\ln{\left[AB9\right]}_{0}-k_{obs}t$$

Indeed, for a second-order kinetic, the considered rate equation (Equation (13)) was:

(13) $$r_{AB9}=-k_{obs}{\left[AB9\right]}^{2}$$

from which Equation (14) was obtained:

(14) $$\frac{1}{\left[AB9\right]}-\frac{1}{{\left[AB9\right]}_{0}}=k_{obs}t$$

**Figure 4:**
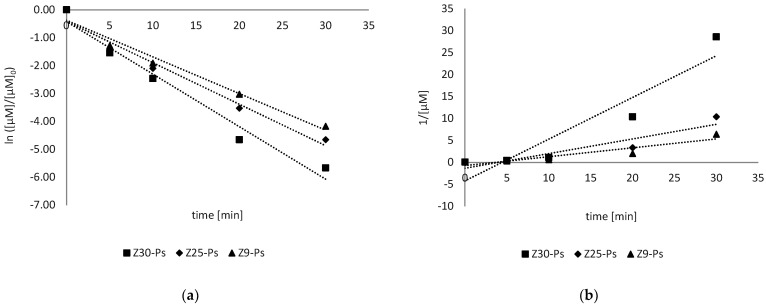
Fitting of the experimental data for Z30-Ps, Z25-Ps and Z9-Ps following the first-order (**a**) and the second-order (**b**) equations. The first-order law better fit the experimental data.

The data obtained were therefore combined with Equations (12) and (14), evaluating the best matching of the experimental data. The best matches were obtained with a first-order equation, in accordance with the literature [5,9,10] (Figure 4).

The R^2^ values obtained for each fitting with different sizes of ZnO were compared and are organized in the Table 1 below:

#### 3.1.2. Degradation of Organic Species with ZnO-Based Photocatalyst: The Determination of Reactive Species

In order to verify the mechanism of photocatalysis and distinguish the contribution of the reactive species, scavengers of radical species are usually used as diagnostic tools; indeed, these scavengers react selectively with persistent or temporary radicals. For example, alcohols, such as isopropanol, methanol and tert-butanol, have been commonly used to estimate the HO^•^-mediated mechanism. The iodide ion is an excellent scavenger that reacts with the h_vb_^+^ and HO^•^ adsorbed because h_vb_^+^ can be easily captured by I^−^ (an electron donor) and the oxidation of I^−^ by superficial HO^•^ is also possible.

**Figure 5:**
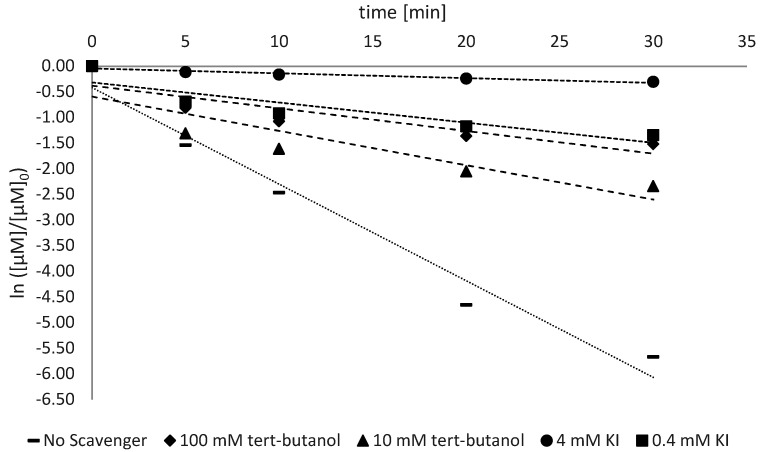
Trend of first-order kinetics following selective inhibitions with tert-butanol and KI. Values refer to Z30-Ps.

**Figure 6:**
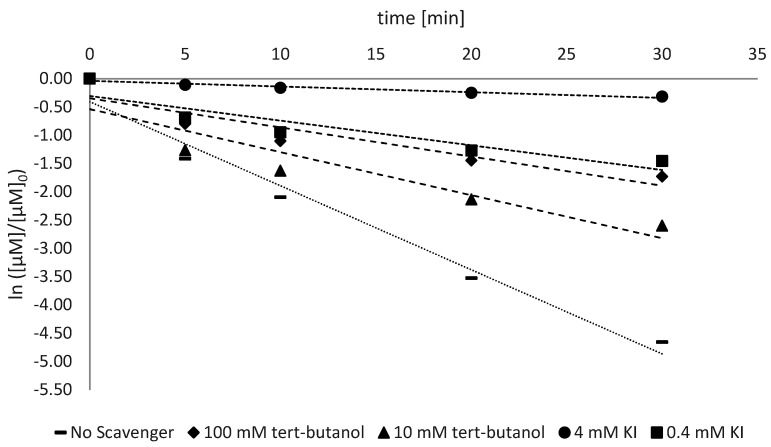
Trend of first-order kinetics following selective inhibitions with tert-Butanol and KI. Values refer to Z25-Ps.

**Figure 7:**
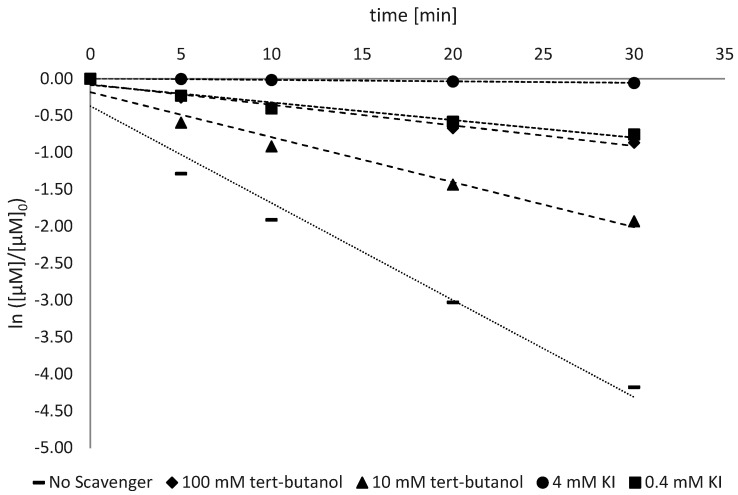
Trend of first-order kinetics following selective inhibitions with tert-Butanol and KI. Values refer to Z9-Ps.

The investigation of the radical activity was conducted as described in Section 2.2.2. The detection of the reactive species was conducted for all the types of ZnO analyzed and the trend is shown in Figure 5, Figure 6 and Figure 7.

In each ZnO analyzed, the values of different k_obs_ obtained were compared. The data obtained are shown in Table 2.

#### 3.1.3. Determination of the Regimen of the Photocatalyzed Reaction by ZnO

The photocatalysis of most organic molecules is described by the Langmuir–Hinshelwood (L–H) model. The experimental data have been rationalized in terms of a modified form of the L–H kinetic model to describe the solid–liquid reaction successfully. Indeed, considering a generic monomolecular reaction (Equation (15)) catalyzed by a porous solid, such as ZnO:

(15) A⇆P

three different steps could be considered in the chemical regime (Equations (16)–(18)):

(16) $$AB9+\sigma ⇆{}_{k1}^{k-1}AB9^{*}$$

(17) $$AB9^{*}⇆{}_{k2}^{k-2}P^{*}$$

(18) $$P^{*}⇆{}_{k3}^{k-3}\sigma +P$$

where σ represents the vacant sites in the catalyst (ZnO) while *AB*9* and P* are the adsorbed Acid Blue 9 and product, respectively.

Experimentally, if the k_obs_ is proportional to the specific surface of different samples (with the surface area as a single difference), it can be concluded that the chemical reaction controls the rate, and the diffusive phenomena are not important. A demonstration of this was obtained by observing the k_obs_ values reported in Table 2, showing a direct proportionality between the rate constant and the surface area.

The experimental data can be rationalized through a modified form of Langmuir–Hinshelwood kinetics [6,12], which have been already successfully used to describe solid–liquid reactions. The surface reaction rate for a unimolecular reaction is proportional to the surface covered, assuming that the reactants are strongly adsorbed on the surface of the catalyst as the products. The effect of the solute concentration in the photocatalysis reaction is therefore given by Equation (19) (assuming the control given by the surface reaction):

(19) $$-r_{0}=\frac{k_{2}K_{AB9}C_{0}}{1+K_{AB9}C_{0}}$$

where *k*_2_ is the rate constant of the surface reaction, *K*_AB9_ is the adsorption coefficient of AB9 on the surface and *C*_0_ is the initial concentration of the dye. At an initial concentration (*C*_0_), the Langmuir–Hinshelwood equation can be modified into Equation (20):

(20) $$-\frac{1}{r_{0}}=\frac{1}{k_{2}K_{AB9}C_{0}}+\frac{1}{k_{2}}$$

**Figure 8:**
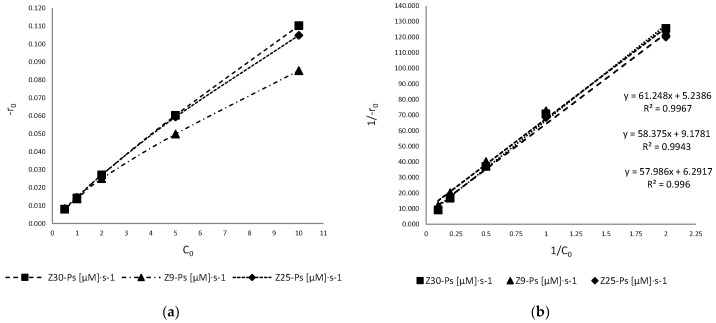
Trend of the initial speeds against the concentration of dye (**a**,**b**). The lines slope shows an inverse relationship with ZnO particle size and surface area.

The applicability of Equation (19) is confirmed by the ratio of *C*_0_ to the initial speeds. Furthermore, in Figure 8b, we can see the correlation coefficient is nearly one, so we can conclude that the photodegradation reaction followed Langmuir–Hinshelwood kinetics.

In the Figure 8b, Equation (20) is plotted, and the linearity obtained shows that the reaction occurs at ZnO’s surface, where the organic species as well as the HO^•^ radicals generated by catalyst UV irradiation are adsorbed [13]. The intersection of this straight line with the ordinate results in 1/k_2_. The adsorption equilibrium constant K_AB9_ was calculated from the slope.

**Figure 9:**
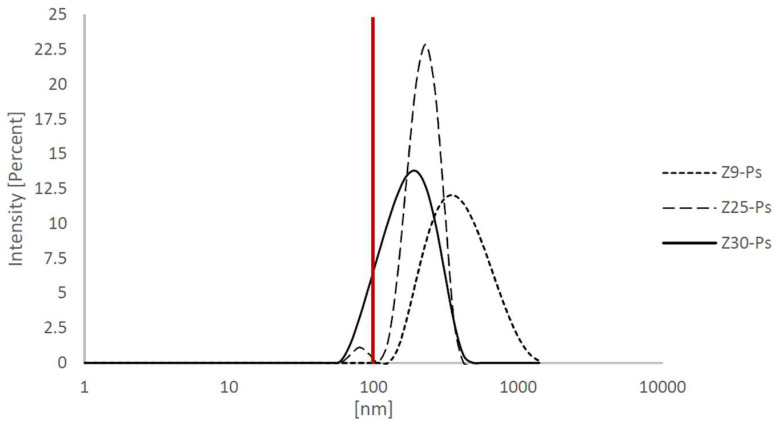
Normal distribution of particle size. The proportions of Z30-Ps’ and Z25-Ps’ nanometric particles can explain the higher photocatalytic activity of these species.

The kinetic constants obtained with the L–H model are compared with the particle sizes of the ZnO and reported in the table below (Table 3):

It is interesting to notice that, with the L–H model, the surface area and particle size influence the rate of the various initial concentrations, like a standard heterogeneous catalyst [14,15,16]. Another point of interest is the normal distribution of the particle size; indeed, the Z30-Ps, which show a higher photocatalytic activity, also show a fraction of their particles in the nanometric region (Figure 9). Additionally, Z25-Ps show a small peak in the nanometric region.

#### 3.1.4. Determination of the Photocatalytic Activity of ZnO against DHHB and EHT

The photocatalysis of two important sunscreen molecules, DHHB and EHT, was also investigated. These kinds of sunscreen were chosen due to the restrictions in different locations, including Hawaii, the U.S. Virgin Islands and Palau. This approach led to banning the widely used oxybenzone and octinoxate [17], while promoting the use of inorganic filters, such as zinc oxide [18]. Further parameters considered for the filter choice were the absorbance maximum wavelength and hydrophilic properties (Table 4).

**Figure 10:**
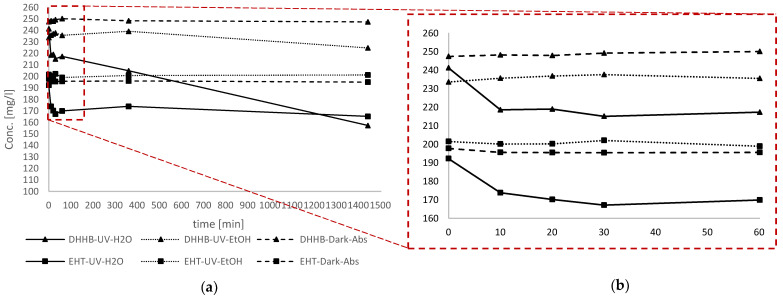
Trend of DHHB and ETH concentration under three different conditions. Irradiation in aqueous solvent with HPS as solubilizer, irradiation in organic solvent (EtOH) and adsorption without irradiation in aqueous solvent with solubilizer. ZnO with a higher surface area (30 m^2^/g) was used for this analysis. (**a**) general trend in 24h, (**b**) focus from t_0_ to 60 minutes.

As described previously, these molecules were solubilized in water through the use of Coceth-7, PPG-1-PEG-9 Lauryl Glycol Ether and PEG-40 Hydrogenated Castor Oil. This procedure allowed us to simulate their behavior in cosmetics emulsions after dispersion in water following topical applications. The degradation of the molecules in water was subsequently measured via HPLC after UV irradiation. The surface absorption of the organic molecules on ZnO was also evaluated by the titration of UV filters over 24 h after dark storage. In addition, the role of water in photocatalysis was evaluated by monitoring the EHT and DHHB photodegradation in two different solvents: water and ethanol. Figure 10 summarizes the results obtained after EHT and DHHB titration in 24 h under different experimental conditions.

## 4. Discussion

Sunscreens are formulations that work by interacting with UV radiation, reflecting or absorbing energetic sunrays. In this process, the energy of UV radiation may induce structural changes in the UV sunscreen filters with possible side interactions with other ingredients selected to achieve optimal protection. In this work, the photocatalytic activity of ZnO with different surface areas was investigated by studying: (a) the order of the reaction; (b) the photocatalytic mechanism most involved in the degradation; (c) the activity of zinc oxide; and finally, (d) the regime that governs the activity of the catalyst (chemical or diffusive).

Regarding the order of the reaction, a first-reaction order was observed to be substantially independent from the surface area. The definition of the reaction order has important consequences for formulations; for example, the half-life depends on it. The first order is substantially independent from the amount of initial reagent, while second-order and zero-order reactions are inversely and directly proportional, respectively, in regard to the initial amount of reagent (sunscreen).

The photocatalytic activity is the most important factor that influences the sunscreen activity of ZnO and TiO_2_ [19]. Like any other photocatalyst, zinc oxide also has two main mechanisms through which its photocatalytic activity is carried out: the formation of the hydroxyl radical by VB and the production of oxygen radical by CB (Figure 1). To assess which of these contributions is the most relevant, a selective inhibition study was conducted with tert-butanol for the radical HO^•^ (with a low affinity with h_vb_^+^) and with the I^−^ ion from KI to capture both h_vb_^+^ (thanks to its electron-donating ability) and HO^•^ radicals (by its oxidation).

The rate constant related to the AB9 degradation showed a decreasing trend from 75% to 95% with 0.4 mM and 4 mM of KI, respectively (considering the average of different ZnO types). This suggests that both HO^•^ and h_vb_^+^ together are responsible for the large degradation of AB9, while other radicals, such as O_2_^•−^, HO_2_^•^ and H_2_O_2_, do not contribute significantly to the dye’s degradation. To determine the relative contribution among HO^•^ and h_vb_^+^, further analyses were performed using tert-butanol as a scavenger for HO^•^ radicals. In Figure 7 (Z9-Ps), the addition of 10 mM and 100 mM of tert-butanol led to a decrease in k_obs_ from 0.0609 min^−1^ to 0.0278 min^−1^, respectively, corresponding to a decrease of 53% and 78% with respect to the control. Comparing the increase in degradation inhibition due to different scavengers, the selective inhibition of HO^•^ turned out to be higher than the inhibition of both HO^•^ and h_vb_^+^. The h_vb_^+^ inhibition for Z9-Ps is very close to those for Z30-Ps and Z25-Ps.

It is believed that the reaction with holes is more important for hydrophobic compounds that are repelled by aqueous solutions and therefore are more likely to undergo adsorption on the surface of the photocatalyst [8].

As for many heterogeneous catalysts, the surface area is a fundamental variable in the reaction rate values. The proportionality between k_obs_ and the surface area is indicative of the presence of a chemical regime, which can be confirmed by studying the initial velocities.

Finally, the photocatalytic capacity of ZnO was tested in conjunction with two organic filters considered not harmful to marine and aquatic environments in general. In recent years, in fact, the trend in cosmetics has been the use of mixed inorganic and organic sunscreen UV filters to reach the best compromise between performance and potential eco-toxicity. However, this may increase potential side effects due to the interaction between the organic and inorganic filters, the latter of these often providing the photocatalytic activity. This trend forces formulators to consciously use filters and have a deeper knowledge of the potential problems involved in the use of these molecules. In order to understand how ZnO can degrade or modify organic filters, the latter were chosen with a different lipophilicity (log P), then solubilized in water with the help of a solubilizer or in pure ethanol and finally irradiated with a UV lamp.

Figure 10 clearly shows that the limited filters decrease with ZnO, with a loss around 0.08% for DHHB and 1.45% for EHT in 24 h. This low but significant difference could be explained by considering the Log P of the filters: the ZnO surface affinity shows an inverse relationship with the molecules’ lipophilicity. In ethanol as a solvent, a low degradation difference between the filters was observed as well, with a reduction of 3.85% for DHHB and 0.14% for EHT. This behavior could be explained by the fact that in ethanol as a solvent, ZnO can acquire hydrophilic properties, thus favoring the absorption of the least lipophilic molecules (DHHB).

In regard to the aqueous environment in which filters end up after their use, we can observe a differing decrease in organic filters after 24 h of exposure, with a 34.77% loss of DHHB and 14.08% loss of EHT. This evidence supports the role of water in ZnO-photoinduced degradation reactions that act via strongly reactive HO^•^ species. This explanation is consistent with what is reported above in the results. Actually, even in ethanol, radical species can occur, as shown by the standard enthalpy of formations [20], but they will be less energetic (Equations (21) and (22)):

(21) $$H_{2}O\to HO^{•}\;\Delta H_{f\;298K}=9.31\;\mathrm{kcal}/\mathrm{mol}$$

(22) $$CH_{3}CH_{2}OH\;\to CH_{3}CH_{2}O^{•}\;\Delta H_{f\;298K}=-2.7\;\mathrm{kcal}/\mathrm{mol}$$

Another possible explanation for the different photocatalytic activity may be found in the presence of water in the ethanol; indeed, if sufficient water is present, the difference in behavior could be explained by the kinetic solvent effect [21].

**Figure 11:**
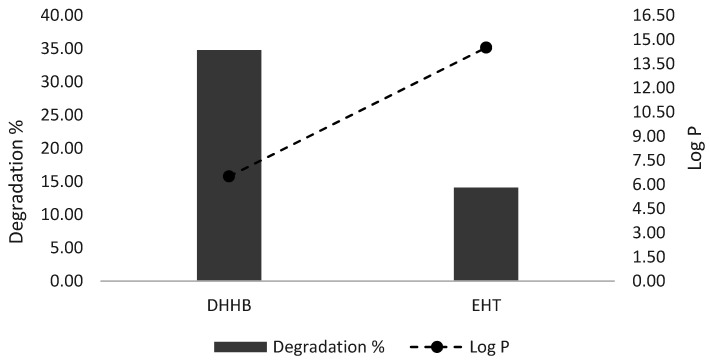
Comparison of DHHB and EHT degradation in relation to Log *p* values. The degradation is directly proportional to the hydrophilicity of the filters.

An inverse relationship was observed between the filters’ degradation and their Log P, as shown in Figure 11.

The greater DHHB hydrophilicity makes the molecules more available for interactions with the hydroxyl radical, while in the case of EHT, these will be more favorably hosted in a surfactant layer, thus preventing a complete interaction.

**Figure 12:**
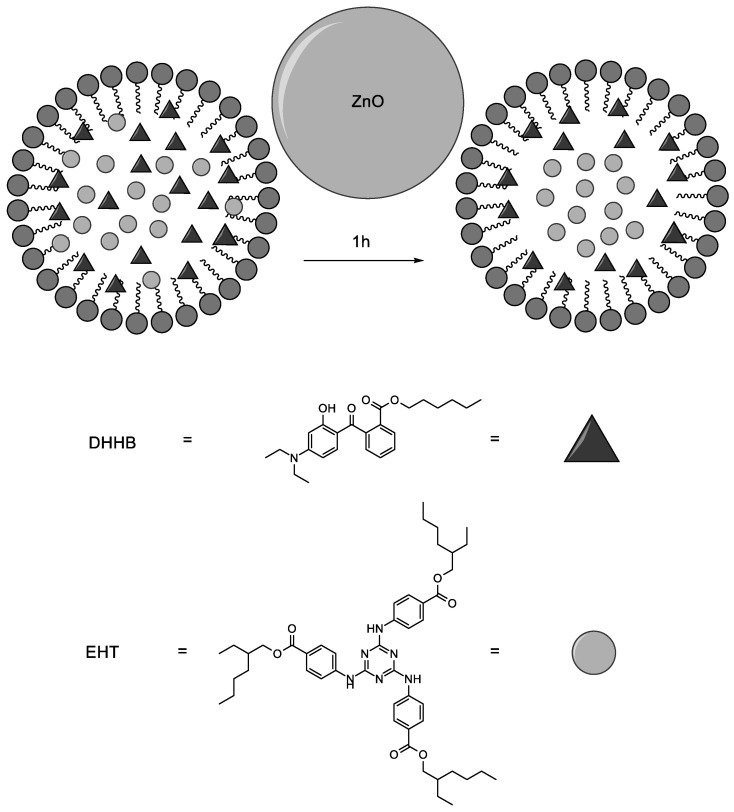
Proposed mechanism to explain the difference in the degradation of filters over 24 h.

Another point of interest is the difference in the degradation trend, which appears very large in the first 60 min and then decreases slowly in the remaining 23 h. This behavior was more pronounced for EHT, in which after an initial fast decrease, a plateau was reached in the following hours. The difference in degradation between DHHB and EHT in the aqueous solvent could be explained by the different position of filter molecules in the micelles [22]. Indeed, the most hydrophilic molecules will be maintained closer to the hydroxylic head of surfactant, while in contrast, the most lipophilic molecules will be stored in the core (bulk) of the micelle. The molecules closer to the water will be also closer to the catalyzer ZnO, so these molecules will undergo faster degradation compared to the lipophilic ones. The faster initial degradation could be due to the consumption of both of them on the surface of the micelles. Figure 12 schematize the described process.

## 5. Conclusions

Using a safe-by-design approach, the photocatalytic activity of ZnO and its possible influence on the degradation of two organic UV sunscreen filters, DHHB and EHT, classified (taken alone) as reef-safe organic molecules, were investigated. An Acid Blue 9 molecule was used as a marker for the degradation kinetics by ZnO. It was determined that the kinetics followed a first-order equation in which the contribution of hydroxyl radicals played a fundamental role. The action of hydroxyl radicals was of extreme importance in regard to the application of ZnO in the cosmetic field, as interactions with water were present both in the early stages of the product’s life (with emulsions containing quantities of water from 40% to 60%) and after the application of the product. In fact, cosmetic emulsions applied to the skin also easily reach the wastewater of cities and accumulate in sedimentation tanks; in this way, they become a primary issue for bacteria, interfering with marine flora and fauna, and causing problems in the purification of domestic waters.

In this work, we focused firstly on studying the degradation kinetics of water-soluble organic molecules and secondly on determining the regime that drives the degradation reaction. The determination of the catalytic regimen (chemical or diffusive) was of interest because it allows us to implement formulation strategies in cosmetic design that can limit the interaction of the ZnO both with the ingredients present and with the skin. Studies have showed that the regime governing AB9 degradation is chemical and that it can be described with a modified form of Langmuir–Hinshelwood kinetics.

Finally, a solution of the two organic UV filters, DHHB and EHT, which have both been presumed to be “non-dangerous” for coral reefs and which have absorption spectra against UVA and UVB, respectively, was prepared to verify the impact of the photocatalytic action. The results showed an inverse proportionality between degradation and Log P with an increased degradation for the most hydrophilic filter. Further confirmation of the ^•^OH photodegradation activity was shown by a comparison between ZnO’s action in aqueous and organic solvents. In ethanol as a solvent, no change in the filter concentration was found after 24 h irradiation. We believe this approach to be very significant in the competition between safe-by-design formulations, in which the possible interactions between filters and between filters and formulations have to be taken into account as early as the design phase in order to limit side effects on human and environmental health.

## Figures and Tables

**Table 1:** Comparison between the R^2^ obtained from the first- and second-order fittings with different particle sizes of ZnO. The values confirm a first-order equation better supports the experimental data. *p* < 0.001 for Z30-Ps and Z25-Ps; 0.01 < *p* < 0.05 for Z9-Ps.

	Z30-Ps	Z25-Ps	Z9-Ps
**R^2^ (I Order)**	0.9709 ± 0.0012	0.9739 ± 0.0015	0.9735 ± 0.0017
**R^2^ (II Order)**	0.8768 ± 0.0022	0.8515 ± 0.0018	0.8588 ± 0.0021

The values of k_obs_ show a better fitting for the first-order reaction [11] with no significant difference between the three different surface areas.

**Table 2:** k_obs_ as a function of the particle sizes of ZnO. Comparison with the various inhibitions. 0.01 < *p* < 0.05 for Z30-Ps vs. Z25-Ps and for Z30-Ps vs. Z9-Ps.

	Rate Constant			Rate Constant Inhibition (%)		
	Z30-Ps	Z25-Ps	Z9-Ps	Z30-Ps	Z25-Ps	Z9-Ps
**No Scavenger**	0.188 ± 0.019	0.1486 ± 0.015	0.1313 ± 0.017	-	-	-
**100 mM tert-butanol**	0.044 ± 0.005	0.0515 ± 0.007	0.0278 ± 0.003	76.53	65.34	78.83
**10 mM tert-butanol**	0.067 ± 0.008	0.0761 ± 0.010	0.0609 ± 0.007	64.47	48.79	53.62
**4 mM KI**	0.009 ± 0.001	0.01 ± 0.001	0.002 ± 0.0001	95.01	93.27	98.48
**0.4 mM KI**	0.039 ± 0.005	0.0436 ± 0.006	0.0238 ± 0.003	79.18	70.66	81.87

**Table 3:** Relation between particle size and kinetic constant. The particle size and related surface area play a significative role in photocatalysis.

	UM	Z30-Ps	Z25-Ps	Z9-Ps
**1/k**	[µM]^−1^·s	5.239	6.292	9.178
**1/kK_AB9_**	[µM]^−1^	61.248	57.986	58.375
**k**	[µM]·s^−1^	0.191	0.159	0.109
**K_AB9_**	[µM]^−1^	0.003	0.003	0.002
**Particle size**	nm	186.3	228.4	425.1

**Table 4:** Main physical properties and structure of DHHB and EHT molecules.

Trade Name	Abbreviation	CAS	INCI	Molecular Structure	Molecular Weight	Molecular Formula	Log P	UV Region/Absorbance Maximum Wavelength [nm]
**Uvinul^®^ A Plus**	DHHB	302776-68-7	diethylamino hydroxybenzoyl hexyl benzoate	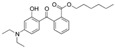	397.5	C_24_H_31_NO_4_	6.5	UVA/354
**Uvinul^®^ T 150**	EHT	88122-99-0	ethylhexyl triazone	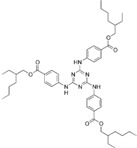	823.1	C_48_H_66_N_6_O_6_	14.5	UVB/314

## Data Availability

Data are contained with the article.
